# Gallic acid antagonizes deoxynivalenol toxicity by inhibiting DON-induced ferroptosis

**DOI:** 10.1038/s41538-026-00782-y

**Published:** 2026-03-05

**Authors:** Haorong Wang, Jiacui Xu, Jinghan Feng, Yifan Zhang, Zixian Zhao, Yan Lv, Chuanqi Wang, Jing Zhang, Yongxing Ai

**Affiliations:** 1 College of Animal Science, Jilin University, Changchun, Jilin China; 2 Jilin Provincial Key Laboratory of Livestock and Poultry Feed and Feeding in Northeastern Frigid Area, Changchun, Jilin China; 3 State Key Laboratory for Diagnosis and Treatment of Severe Zoonotic Infectious Diseases, College of Veterinary Medicine, Jilin University, Changchun, Jilin China; 4 National Grain Industry Technology Innovation Center (Medicinal Functional Resources Development), Academy of National Food and Strategic Reserves Administration, Beijing, China

**Keywords:** Biochemistry, Cell biology, Diseases, Molecular biology

## Abstract

Deoxynivalenol (DON) is a prevalent foodborne mycotoxin severely compromising livestock and poultry health and food safety. This study evaluated gallic acid (GA) —a top-performing candidate among screened polyphenols—in counteracting DON toxicity. In chicken embryo fibroblasts, 0.5 μM DON reduced viability to ~55% and induced oxidative stress, while co-treatment with 160 μM GA restored viability to ~79%. In chicks, DON caused hepatic injury and intestinal villus shortening, effects that GA effectively mitigated. Transcriptomic and biochemical assays revealed that DON triggers ferroptosis by disrupting iron homeostasis and lipid peroxidation elevation, whereas GA activated the Nrf2 pathway and upregulated key cytoprotective genes (*GPX4*, *FTH1*, *SLC7A11*, *HO-1*) to inhibit ferroptosis. These results demonstrate that GA antagonizes DON toxicity primarily through Nrf2-mediated ferroptosis suppression. In summary, this investigation provided not only a mechanistic basis for GA’s standalone application but also a rationale for targeted combinatorial therapy from multi-mechanistic perspectives to alleviate DON-induced damage and safeguard animal health and food safety.

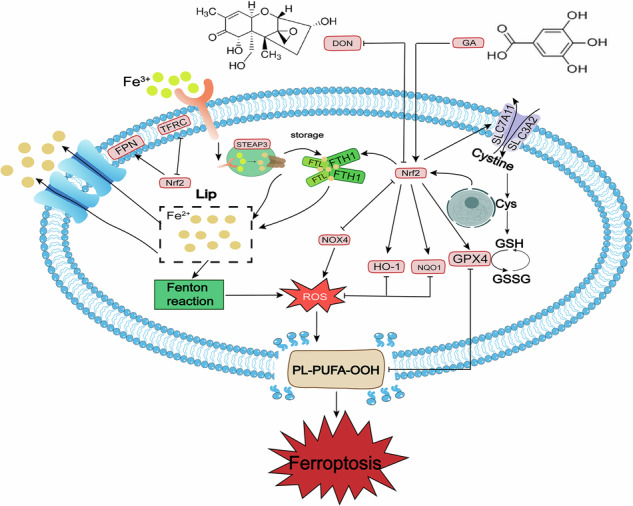

## Introduction

Mycotoxins, toxic secondary metabolites of fungi, globally contaminate food and feed, posing significant threats to human and animal health^1,2^. Among these, DON ranks as one of the five agriculturally critical mycotoxins, compromising the safety of staple crops including corn, barley, and wheat^3,4^. DON contamination is primarily caused by *Fusarium graminearum*, *Fusarium asiaticum*, and related species, which cause *Fusarium* head blight in cereals and ear rot in maize^5^. Beyond field contamination, DON production persists during feed processing, storage, and transportation. Critically, climate change is escalating global DON contamination, urging the development of effective mitigation strategies^6^. Current DON detoxification strategies include physical, chemical, and biological approaches; however, physical and chemical techniques cannot fully eradicate DON, with residuals remaining to endanger human and animal health and compromise food and feed safety^7^. Biological interventions—particularly in vivo therapeutic agents that target DON toxicity to mitigate or eliminate organismal damage—are therefore indispensable. Natural product-based therapies derived from ethnoveterinary medicine, in particular, represent a well-documented and highly promising option due to their low toxicity and accessible raw material sources^8^. DON exerts particularly severe toxicity in monogastric animals^9^. Acute high-dose DON exposure induces gastrointestinal injury—manifested by intestinal inflammation, hemorrhage, salivation, vomiting, and diarrhea—while concurrently causing multi-organ damage, with the liver and intestine being the primary targets^10,11^. This is especially relevant to poultry production: corn constitutes 55% of chick starter diets, rendering chicks highly vulnerable to DON^12,13^. Unmitigated DON toxicity severely impairs growth performance and poses long-term health hazards.

Mechanistically, DON toxicity involves multiple signaling pathways, including Wnt/β-catenin, FOXO, TLR4/NF-κB, Notch1, PI3K/Akt, and JAK/STAT signaling^14^. Beyond these pathways, emerging evidence highlights the critical role of ferroptosis—an iron-dependent form of regulated cell death driven by lipid peroxidation—in DON-induced damage^15,16^. Growing evidence implicates ferroptosis in DON-associated pathology: it mediates intestinal injury in piglets^17^, testicular dysfunction^18^, and hepatic damage via iron dysregulation and mitochondrial impairment^19^. Ferroptosis has been harnessed to induce tumor cell death as a target for antitumor therapies^20^, while cross-disciplinary evidence (e.g., infection-associated oxidative stress contexts) indicates its dysregulation may also exacerbate disease progression and impair prognosis^21^. These findings underscore that ferroptosis— as a core mechanism governing cell fate and the key mediator of DON-induced damage—is a critical target to prioritize when screening for DON antagonists. Furthermore, inhibiting ferroptosis represents one of the most promising strategies for rescuing DON-induced injury^22,23^.

This prompted us to focus on screening natural compounds that can target the ferroptosis pathway, with particular emphasis on those capable of alleviating redox imbalance—the core driver of this cell death process. Based on this rationale, the present study aimed to identify a high-efficacy, low-toxicity agent against DON. We thus screened several natural polyphenols—including gallic acid (GA), caffeic acid (CA), proanthocyanidins (PC), chrysin, and several other polyphenolic compounds—leveraging their well-recognized antioxidant properties. GA was selected for in-depth investigation: as a trihydroxybenzoic acid abundant in gallnuts, tea, and various fruits, it is a well-documented potent antioxidant, anti-inflammatory, and chemopreventive agent^24^. GA has been shown to protect against hepatic steatosis^25^ and intestinal barrier dysfunction^26^. It is a recognized activator of the cytoprotective nuclear factor erythroid 2-related factor 2 (Nrf2) signaling pathway that orchestrates antioxidant gene expression^27^. This property, combined with its direct free radical-scavenging capacity, makes GA a promising candidate to counteract DON-induced redox imbalance. Furthermore, GA alleviates exercise-induced oxidative stress and ferroptosis^28^, providing direct evidence of its ability to modulate this cell death pathway under redox perturbation. This ability to modulate ferroptosis, combined with its established antioxidant properties, further supports GA as a promising candidate to counteract DON-induced toxicity. Recent investigations have advanced our understanding of GA’s bioactivity, highlighting its chemical versatility and emerging applications in advanced formulations^29^. Additionally, the historical use of GA-containing plants in ethnoveterinary practice underscores its suitability as a natural agent for animal health^8^.

Notably, layer chicks, a representative monogastric livestock species, face elevated mycotoxin exposure risk and heightened vulnerability to such toxicity due to the high grain proportion in their diet^9,13,30^. DON-induced damage during the early developmental stage of chicks can profoundly affect growth, health, and future production performance, making the mitigation of DON toxicity in chicks of particular practical importance^9,12,13^. The chick model was therefore selected for in vivo evaluation. Compared to differentiated cells, chicken embryo fibroblasts (CEFs) cells exhibit greater susceptibility to DON^31^ and are widely used in pharmacological and mycotoxin studies^32^.

Based on the implication of redox imbalance and ferroptosis in DON pathology^22^, and GA’s profile as an Nrf2 activator^27^ with anti-ferroptotic potential^28^, we hypothesized that GA protects against DON toxicity by enhancing cellular antioxidant defenses via the Nrf2 pathway, thereby suppressing ferroptosis. To test this central hypothesis, the present study set three specific objectives: (1) Optimize the administration conditions of GA and validate its efficacy in antagonizing DON toxicity in chicken cells; (2) Verify the protective effect of GA against DON-induced hepatointestinal damage in layer chicks using pathological and biochemical assays; (3) Elucidate the toxicity mechanism of DON (focusing on oxidative stress and ferroptosis) and the antagonistic mechanism of GA via omics and biochemical analyses, including changes in related signaling pathways, gene expression, and enzyme activities.

## Materials and methods

### Cell culture

CEF cells were isolated as previously described^33^. Cells were cultured in Dulbecco’s Modified Eagle Medium (DMEM) supplemented with 10% (v/v) fetal bovine serum (FBS) at 37 °C in a humidified atmosphere containing 5% CO₂.

### Animal experiments

All animal procedures were approved by the Institutional Animal Care and Use Committee (IACUC) of Jilin University (Approval No. SY202503061). Thirty 1-day-old healthy Jingbai No.1 chicks (Huayu Agricultural Technology Co., Ltd, Changchun, China) were randomly divided into three groups (Control group, DON group, DON + GA group; *n* = 10/group). The Control group was fed a basal diet (Zhengda Yongji Industry Co., Ltd., Jilin, China). The DON group was fed the basal diet supplemented with *F. graminearum*-fermented corn, achieving a final DON concentration of 5 mg/kg feed^34^. This DON dosage is consistent with real-world dietary DON contamination levels in affected regions, aligned with established models for inducing subacute toxicity in poultry^34^, and validated in our preliminary experiments to reliably induce reversible pathological changes suitable for a rescue study design. The DON + GA group received the same DON-contaminated diet as the DON group, with daily oral gavage of 1 mL GA solution (100 mg/kg body weight^35^) (Shanghai Yuanye Bio-Technology Co., Ltd, Shanghai, China). The selected GA dosage falls within the range of safe dietary supplementation levels reported for poultry, based on its well-documented safety and efficacy profile in prior studies^35^, and further confirmed feasible in our pilot experiments. The *F. graminearum*-fermented corn was prepared by incubating corn with *Fusarium graminearum* (donated by Professor Xianghui Zhang, College of Plant Science, Jilin University^36^) at 25 °C. The DON content in fermented corn was quantified by the National Feed Quality Inspection and Testing Center (Beijing, China), Institute of Quality Standards and Testing Technology for Agro-products, Chinese Academy of Agricultural Sciences. Chicks were housed under controlled environmental conditions with ad libitum access to feed and water. After 7 days of treatment, chicks were euthanized by exsanguination following anesthesia.

### Cell viability assay

DON (≥98%, Shanghai Jizhi Biochemical Technology Co., Ltd., China) was dissolved in dimethyl sulfoxide (DMSO) to prepare a 10 mM stock solution. CA, PC, and GA (Shanghai Yuanye Bio-Technology Co., Ltd.) were dissolved in phosphate-buffered saline (PBS) to prepare 50 mM stock solutions. A DON concentration of 0.5 μM was selected to establish a sub-lethal injury model, which reduced cell viability by approximately 50%–60% and provided a robust and reproducible window for evaluating cytoprotective agents. CEF cells (8000/well) were seeded in 96-well plates, cultured for 12 h, then treated with DON and/or GA for 9 h. Cell viability was assessed using CCK-8 kit (10% v/v; Meilun Biotechnology, Dalian, China) after incubation at 37 °C for 1 h. Absorbance at 450 nm was measured using a microplate reader (TECAN, Männedorf, Switzerland).

### Intracellular ROS detection

Intracellular reactive oxygen species (ROS) levels were detected according to the instructions of the ROS Detection Kit (Beyotime Biotechnology, Shanghai, China) after cell treatment. Briefly, CEF cells (8 × 10⁵ cells/well) were seeded in 6-well plates and treated with 0.5 μM DON alone or 0.5 μM DON plus 160 μM GA for 9 h. Cells were incubated with 10 μM DCFH-DA at 37 °C for 20 min, washed twice with serum-free DMEM, and observed under a fluorescence microscope (OLYMPUS IX71, Tokyo, Japan). DCF fluorescence (green, indicator of ROS) was captured at excitation/emission wavelengths of 488/525 nm. The average fluorescence intensity of each group was quantified using ImageJ2 software (National Institutes of Health, Bethesda, MD, USA)^37^.

### Analysis of oxidative stress indicators

For cellular analysis, CEF cells were seeded in 6-well plates at a density of 8 × 10⁵ cells per well. After treatment, cells were harvested by scraping and lysed in ice-cold lysis buffer via ultrasonication. For tissue analysis, approximately 0.5 g of liver tissue was homogenized in 4.5 mL of ice-cold normal saline (0.9% NaCl, 1:9 w/v) using a sterilized mortar on ice. Cell lysates and tissue homogenates were processed according to the respective kit protocols. Samples for superoxide dismutase (SOD) activity and total antioxidant capacity (T-AOC) assays were centrifuged at 12,000 × *g* for 5 min at 4 °C. Samples for catalase (CAT) activity were centrifuged at 4000 × *g* for 10 min at 4 °C. Samples for reduced glutathione (GSH) content determination were centrifuged at 3500 × *g* for 10 min at 4 °C. The supernatants were collected for subsequent analyses.

All assays were performed using commercial kits following the manufacturers’ instructions: Total SOD Activity Detection Kit (WST-8 method; Beyotime Biotechnology), Catalase Assay Kit (Nanjing Jiancheng Bioengineering Institute), Reduced Glutathione Assay Kit (Nanjing Jiancheng Bioengineering Institute), Total Antioxidant Capacity Assay Kit (Nanjing Jiancheng Bioengineering Institute). Protein concentration of each sample was quantified with a Bradford Protein Assay Kit (Beyotime Biotechnology). Final indices were expressed as follows: SOD activity (U/μg protein), CAT activity (U/mg protein), GSH content (μmol/g protein), and T-AOC (mmol/g protein).

### Histopathological examination

Tissue morphological changes were evaluated by hematoxylin and eosin (H&E) staining. Briefly, liver and intestinal tissues were fixed in 4% (w/v) paraformaldehyde at room temperature for 24 h, followed by dehydration through a graded ethanol series (70%, 80%, 90%, 95%, and 100% v/v), clearing with xylene, and embedding in paraffin. Paraffin-embedded tissues were sectioned into 3-μm-thick slices using a microtome, mounted on glass slides, stained with hematoxylin (5 min) and eosin (3 min), dehydrated, cleared with xylene, and mounted with neutral balsam. Histological images were captured using an optical microscope (Nikon DS-U3, Tokyo, Japan). Intestinal villus height (from the crypt base to the villus tip) and crypt depth (from the muscularis mucosae to the villus base) were quantified using ImageJ software, with three randomly selected fields per section and five sections per sample.

### Liver function tests

Blood samples were collected from the heart of chicks under ether anesthesia, allowed to clot at room temperature for 30 min, and centrifuged at 1200 × *g* for 10 min at 4 °C to separate serum. Levels of alanine transaminase (ALT), aspartate aminotransferase (AST), γ-glutamyl transpeptidase (γ-GT), and lactate dehydrogenase (LDH) were measured using a fully automated biochemical analyzer (Mindray BS-2000M, Shenzhen Mindray Animal Medical Technology Co., Ltd., Shenzhen, China) with manufacturer-provided kits (IFCC Method for all indices) following standard protocols.

### Liver tissue ROS detection

Liver tissue sections were incubated in 10 μM dihydroethidium (DHE; Invitrogen, Thermo Fisher Scientific, Waltham, MA, USA) diluted in PBS (PBS∶DHE = 200:1, v/v) at 37 °C in the dark for 30 min to label superoxide anions, followed by three washes with PBS. Sections were counterstained with 1 μg/mL DAPI (Beyotime Biotechnology) at room temperature in the dark for 10 min to visualize cell nuclei, washed three times with PBS, and mounted with anti-fade mounting medium (Beyotime Biotechnology). Fluorescence images were acquired using a fluorescence microscope (Nikon Eclipse TI-SR, Tokyo, Japan) with excitation/emission wavelengths of 535/610 nm (DHE, red fluorescence) and 358/420 nm (DAPI, blue fluorescence).

### Transcriptomics analysis

CEF cells (8 × 10⁵ cells/well) were seeded in 6-well plates and cultured overnight, then treated with 0.5 μM DON alone or in combination with 160 μM GA for 9 h. Total RNA was isolated using TRIzol™ Reagent (Invitrogen, USA) according to the manufacturer’s protocol. RNA libraries were constructed and sequenced on the Illumina NovaSeq X Plus platform (paired-end 150 bp reads). Raw sequencing reads were quality-controlled and adapter-trimmed using fastp (v0.23.4) to generate clean reads. Transcript abundance was quantified using RSEM (v1.3.3) with alignment to the *Gallus gallus* reference genome (GRCg6a). Differential gene expression (DEG) analysis was performed using DEGseq (v1.52.2) with thresholds of |log₂(fold change)| > 1 and adjusted *p*-value < 0.05. Gene Ontology (GO) enrichment analysis of DEGs was conducted using Goatools (v1.3.0), and KEGG pathway analysis was implemented using custom R scripts (v4.3.1) with the clusterProfiler package.

### Peroxylipid tests in liver tissue

Malondialdehyde (MDA) levels, a marker of lipid peroxidation, were quantified in liver homogenates using a Lipid Oxidation (MDA) Assay Kit (Beyotime Biotechnology). Briefly, liver homogenates were processed according to the kit instructions, absorbance was measured at 532 nm, and MDA concentration was calculated and expressed as nmol/mg of protein.

For in situ visualization of lipid peroxidation, frozen liver sections were gradually rewarmed to room temperature, incubated with 5 µM BODIPY™ 581/591 C11 probe (Thermo Fisher) diluted in serum-free DMEM at 37 °C in the dark for 20 min, and washes three times with PBS. Nuclei were counterstained with 1 µg/mL DAPI for 2 min, washed, and mounted with anti-fade mounting medium. Fluorescence images were captured using a fluorescence microscope (Nikon Eclipse Ti2, Tokyo, Japan) with excitation/emission wavelengths of 485/515 nm (oxidized BODIPY, green fluorescence) and 358/420 nm (DAPI, blue fluorescence). Green fluorescence intensity was quantified using ImageJ software reflect relative lipid peroxidation level.

### Quantitative analysis of key gene expression levels

Total RNA from CEF cells was extracted using TRIzol™ Reagent. RNA (1 µg total) was reverse-transcribed into cDNA using the All-In-One 5× RT MasterMix Kit (Applied Biological Materials Inc., Canada). Quantitative real-time PCR (qRT-PCR) reactions were performed in triplicate using BlasTaq™ 2× qPCR MasterMix (ABM) on a Bioer LineGene 9600 QPCR System (Hangzhou Bioer Technology, China). Cycling conditions were as follows: 95 °C for 3 min; 40 cycles of denaturation at 95 °C for 10 s and annealing/extension at 60 °C for 30 s. A melting curve analysis was performed to verify primer specificity. Gene expression levels were normalized to β-actin and calculated using the 2^−ΔΔCt^ method. Primer sequences are provided in Supplementary Table S1.

### Liver tissue iron quantification

Liver tissue was accurately weighed and homogenized in ice-cold normal saline (1:9 w/v) using a mortar under an ice-water bath. Homogenates were centrifuged at 2500 × *g* for 10 min at 4 °C, and supernatants were collected for analysis using an Iron Assay Kit (Nanjing Jiancheng Bioengineering Institute). Briefly, 500 µL supernatant was mixed with chromogenic reagent, incubated at 95 °C for 5 min, cooled to room temperature, and absorbance was measured at 520 nm using a microplate reader (TECAN). Total iron concentration in liver tissues was calculated using Formula (1).

1 $${\rm{Iron}}\; {\rm{concentration}}=\frac{{\rm{Sample}}\; {\rm{OD}}-{\rm{Blank}}\; {\rm{OD}}}{{\rm{Standard}}\;{\rm{OD}}-{\rm{Blank}}\; {\rm{OD}}}\times {\rm{Standard}}\;{\rm{concentration}}\left(20\, \mu {\rm{mol}}/{\rm{L}}\right)/{\rm{Protein}}\,{\rm{concentration}}$$

### Mitochondrial membrane potential (MMP) assay

MMP was assessed using the JC-1 Mitochondrial Membrane Potential Assay Kit (Beyotime Biotechnology). CEF cells were incubated with 10 µg/mL JC-1 at 37 °C for 20 min, washed twice with JC-1 buffer, and immediately imaged under a Nikon Eclipse Ti-SR fluorescence microscope. JC-1 aggregates (high MMP, red fluorescence) and monomers (low MMP, green fluorescence) were detected at excitation/emission of 525/590 nm and 490/530 nm, respectively. MMP changes were quantified as the ratio of red to green fluorescence intensity.

### Validation of ferroptosis modulation

Erastin and Ferrostatin-1 (Shanghai Yuanye Bio-Technology Co., Ltd, China) were dissolved in DMSO to prepare 10 mM and 1 mM stock solutions, respectively. Working solutions were diluted in complete DMEM. CEF cells were treated with respective reagents in designated groups for 9 h; solvent controls received an equivalent volume of DMSO (final concentration < 0.01%, v/v, non-toxic to cells).

### Statistical analysis

Data are expressed as mean ± standard error of the mean (SEM). Inter-group differences were analyzed by one-way analysis of variance (ANOVA) followed by Tukey’s post hoc test using GraphPad Prism 9.5 (GraphPad Software, San Diego, CA, USA). Statistical significance was set at *p < 0.05, **p < 0.01, ***p < 0.001.

### Ethical approval

All experimental procedures involving animals were approved by the Institutional Animal Care and Use Committee of Jilin University (Approval No. SY202503061).

## Results

### GA alleviates DON-induced cytotoxicity and oxidative stress in CEF cells

**Fig. 1:**
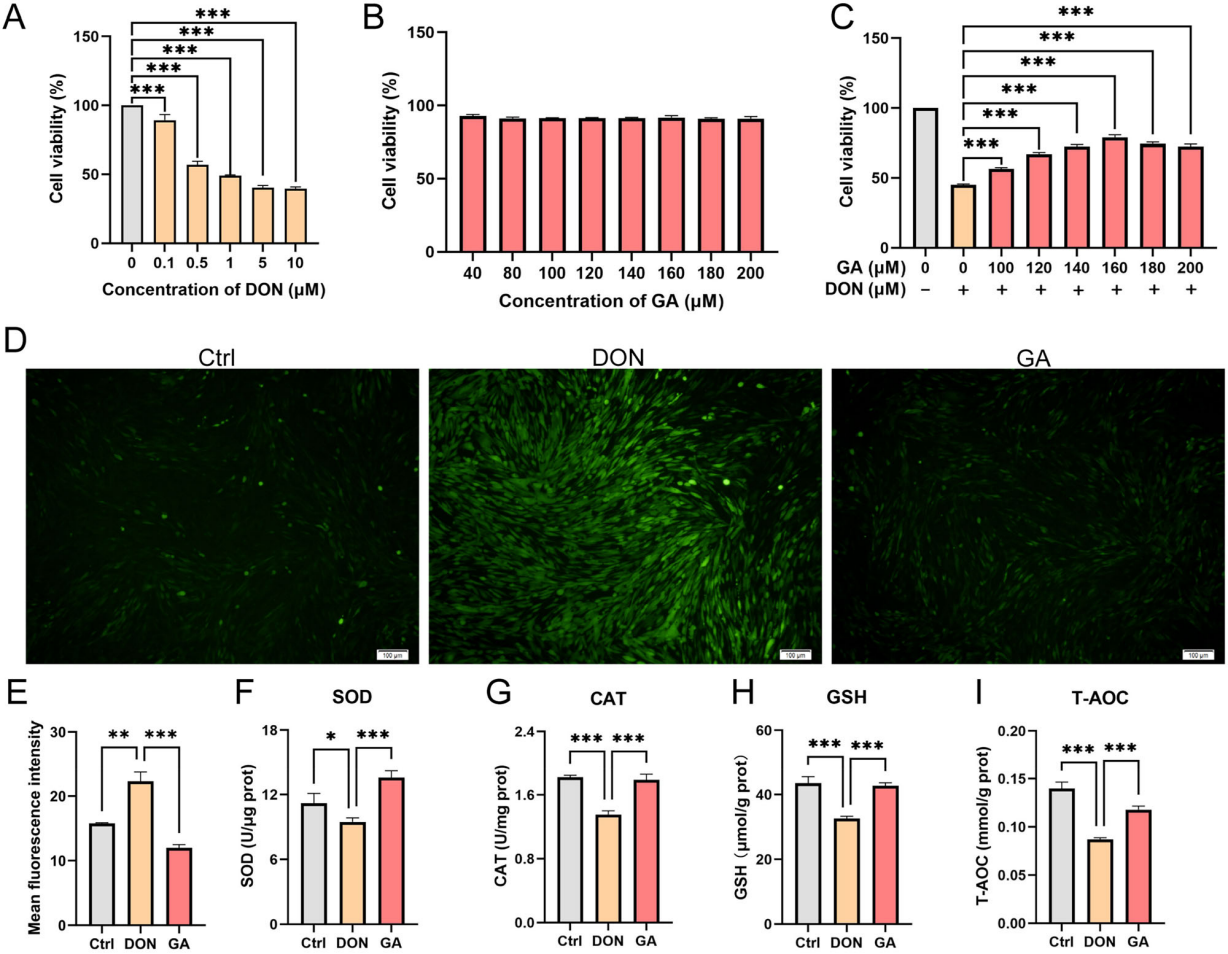
GA mitigates DON-induced cytotoxicity and oxidative stress in CEF cells. **A** Cell viability following 9 h exposure to DON (0.1–10 μM). **B** Cell viability after 9 h treatment with GA (40–200 μM). **C** Restoration of cell viability by co-treatment with GA (100–200 μM) and 0.5 μM DON. **D** Representative fluorescence images of intracellular ROS (detected using a 5 μM DCFH-DA probe; scale bar: 100 μm). **E** Quantification of DCF fluorescence intensity. **F** Intracellular SOD activity (U/μg protein). **G** Intracellular CAT activity (U/mg protein). **H** Intracellular GSH levels (μmol/g protein). **I** Intracellular T-AOC (mmol/g protein). Error bars represent the standard deviations from three independent replicates. Statistical significance: *p < 0.05, ** *p* < 0.01, *** *p* < 0.001 (vs. control or DON group).

Exposure of CEF cells to DON at concentrations ranging from 0.1 to 10 μM for 9 h resulted in a concentration-dependent decrease in cell viability (Fig. 1A). The half-maximal inhibitory concentration (IC₅₀) of DON in CEF cells was calculated as approximately 1.132 μM via non-linear regression analysis of the dose-response curve. A DON concentration of 0.5 μM—which reduced cell viability to approximately 50%–60%, below the IC₅₀ and thus suitable for establishing a sub-lethal injury model—was selected for subsequent rescue experiments. Among the screened polyphenols, GA at a concentration of 40–200 μM exhibited no inherent cytotoxicity (*p* > 0.05) (Fig. 1B). In contrast, CA and PC induced significant cytotoxicity at concentrations above 50 μM (Fig. S1A, B). Co-treatment with GA (100–200 μM) and 0.5 μM DON restored CEF cell viability in a dose-dependent manner compared to the DON-alone group (*p* < 0.001). Maximal protection (78.97% cell viability) was achieved at a GA concentration of 160 μM (Fig. 1C). By comparison, CA and PC only restored cell viability to only 70.42% and 65.37%, respectively (Fig. S1C, D). Given its non-toxic profile and superior protective effect, 160 μM GA was selected for subsequent experiments (Fig. 1C).

DON exposure significantly induced oxidative stress in CEF cells, as evidenced by a marked increase in intracellular ROS accumulation (reflected by green fluorescence intensity, *p* < 0.01) (Fig. 1D, E). The activities of key antioxidant enzymes, SOD and CAT, were significantly suppressed by DON treatment (*p* < 0.05 and *p* < 0.01, respectively), but were restored in the DON+ GA co-treatment group (*p* < 0.001) (Fig. 1F, G). The non-enzymatic antioxidant GSH was significantly depleted in DON treated cells (*p* < 0.001), while GA co-treatment replenished intracellular GSH levels (*p* < 0.001) (Fig. 1H). T-AOC followed a similar trend: DON-induced suppression of T-AOC (*p* < 0.001) was reversed by GA co-treatment (Fig. 1I). Collectively, these results indicate that DON induces cytotoxicity in CEF cells through oxidative stress mechanisms, including elevated ROS production, depletion of GSH and T-AOC, and impairment of SOD/CAT activities. GA comprehensively counteracts these perturbations, which explains its cytoprotective effect.

### GA mitigates DON-induced multi-tissue damage and oxidative stress in layer chicks

**Fig. 2:**
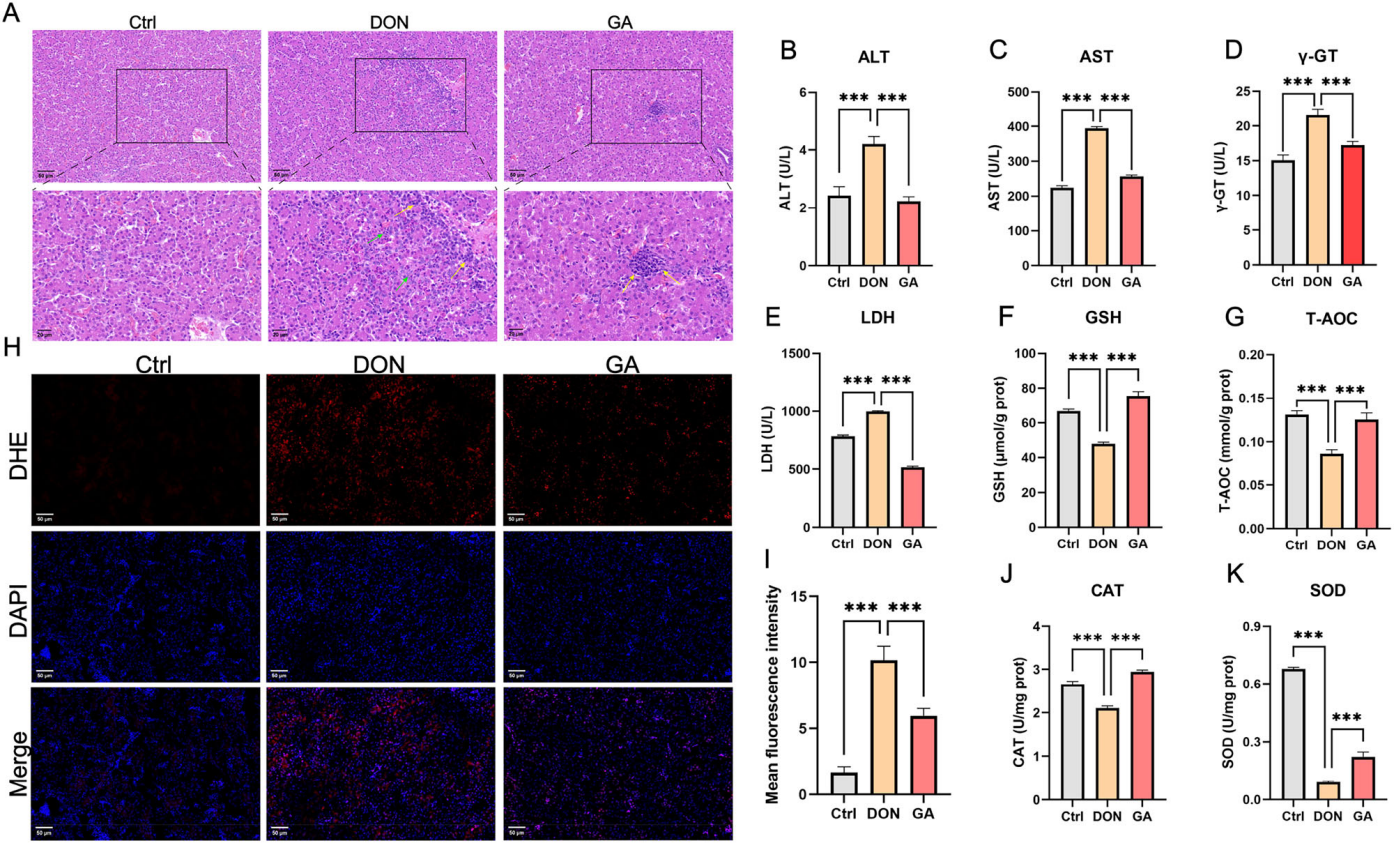
GA alleviates DON-induced liver tissue damage and oxidative stress in layer chicks. **A** H&E staining of hepatic tissues. Yellow arrows indicate inflammatory cell infiltration; green arrows indicate necrotic areas. **B** Serum alanine transaminase (ALT) level. **C** Serum aspartate aminotransferase (AST) level. **D** Serum γ-glutamyl transpeptidase (γ-GT) level. **E** Serum lactate dehydrogenase (LDH) level. **F** Tissue glutathione (GSH) level. **G** Tissue total antioxidant capacity (T-AOC). **H** DHE fluorescence staining for detection of tissue ROS levels (red fluorescence intensity reflects intracellular superoxide anion accumulation). **I** Quantitative analysis of red fluorescence intensity in (**H**). **J** Tissue catalase (CAT) activity. **K** Tissue superoxide dismutase (SOD) activity. Error bars represent the standard deviation from three independent replicates. Statistical significance: *p < 0.05, ** *p* < 0.01, *** *p* < 0.001 (vs. control or DON group).

Histopathological analysis revealed that DON exposure caused severe liver damage in layer chicks, characterized by disorganized hepatocyte arrangement, indistinct hepatic plates, sinusoidal narrowing, portal fibrosis, and focal inflammatory cell infiltration (Fig. 2A). In contrast, chicks treated with GA exhibited regular hepatocyte arrangement, a clear hepatic plate structure, no hepatocyte necrosis, and only partial inflammatory cell infiltration (Fig. 2A). Serum biomarkers of hepatotoxicity including ALT, AST, γ-GT, and LDH, were significantly elevated in DON-treated chicks. GA treatment significantly attenuated these increases (*p* < 0.001) (Fig. 2B–E), confirming that GA alleviates DON-induced liver injury. Detection of antioxidant indices showed that DON exposure reduced tissue GSH levels, T-AOC capacity, and CAT/SOD activities while increasing ROS production, indicating DON-induced oxidative stress in liver tissue. GA treatment significantly reversed these changes, demonstrating its protective effect against DON-induced tissue oxidative stress (Fig. 2G–K).

**Fig. 3:**
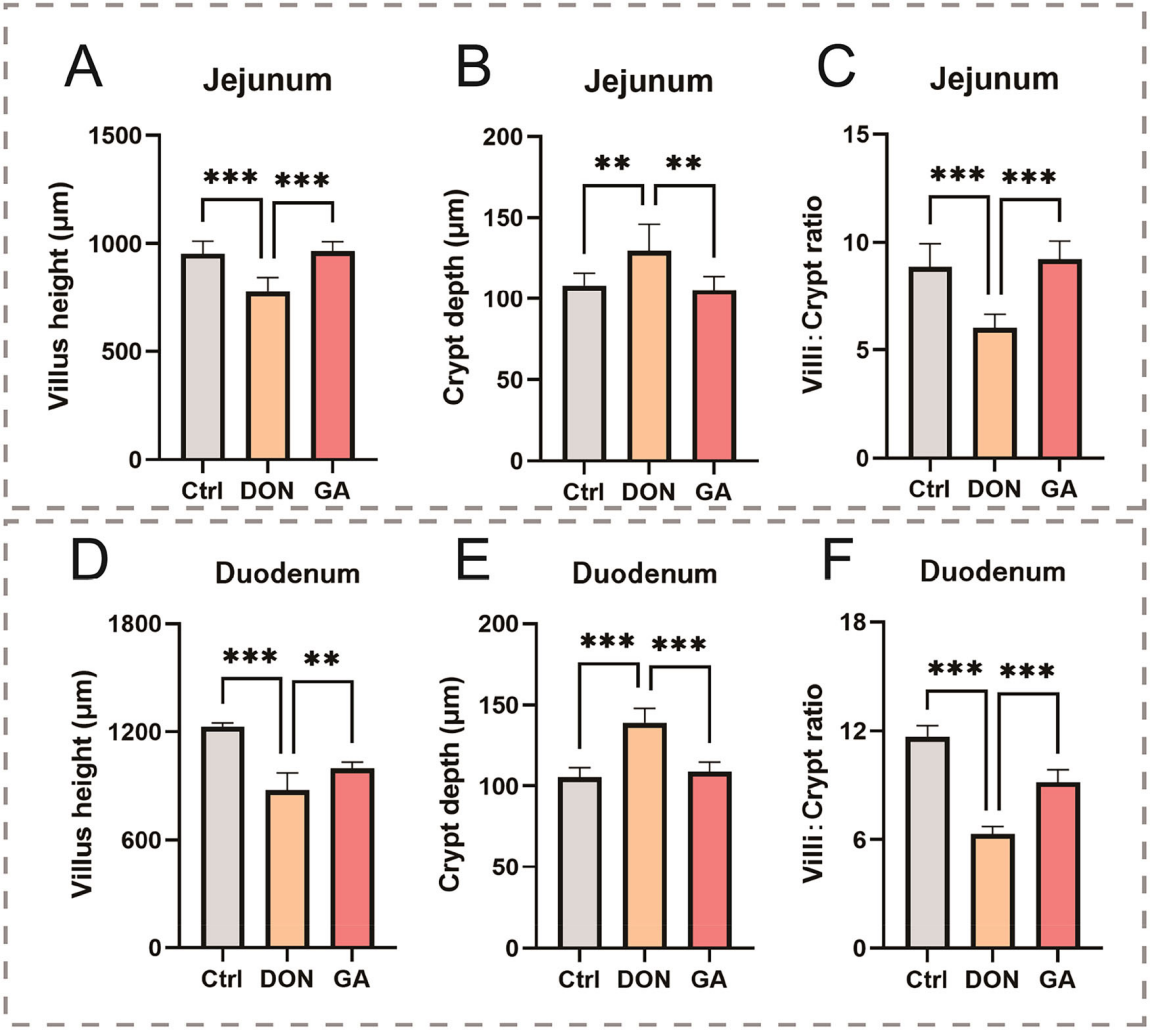
Gallic acid ameliorates DON-induced intestinal morphometric damage in chicks. **A** Intestinal villus height in jejunum. **B** Intestinal crypt depth in jejunum. **C** Ratio of intestinal villus height and crypt depth in the jejunum. **D** Intestinal villus height in the duodenum. **E** Intestinal crypt depth in the duodenum. **F** Ratio of intestinal villus height and crypt depth in the duodenum. Error bars represent the standard deviations from three independent replicates. Statistical significance: *p < 0.05, ** *p* < 0.01, *** *p* < 0.001 (vs. control or DON group).

Although no overt pathological damage was observed in H&E-stained sections of duodenum and jejunum (Fig. S2), DON exposure induced significant morphometric alterations: intestinal villus height was significantly decreased (*p* < 0.001), crypt depth was significantly increased (*p* < 0.001), and the villus height/crypt depth ratio was significantly reduced (*p* < 0.001). Co-treatment with GA significantly normalized these morphometric changes (*p* < 0.001), indicating alleviation of DON-induced intestinal damage (Fig. 3).

### Transcriptomic insights into the mechanisms of GA protection against DON toxicity

**Fig. 4:**
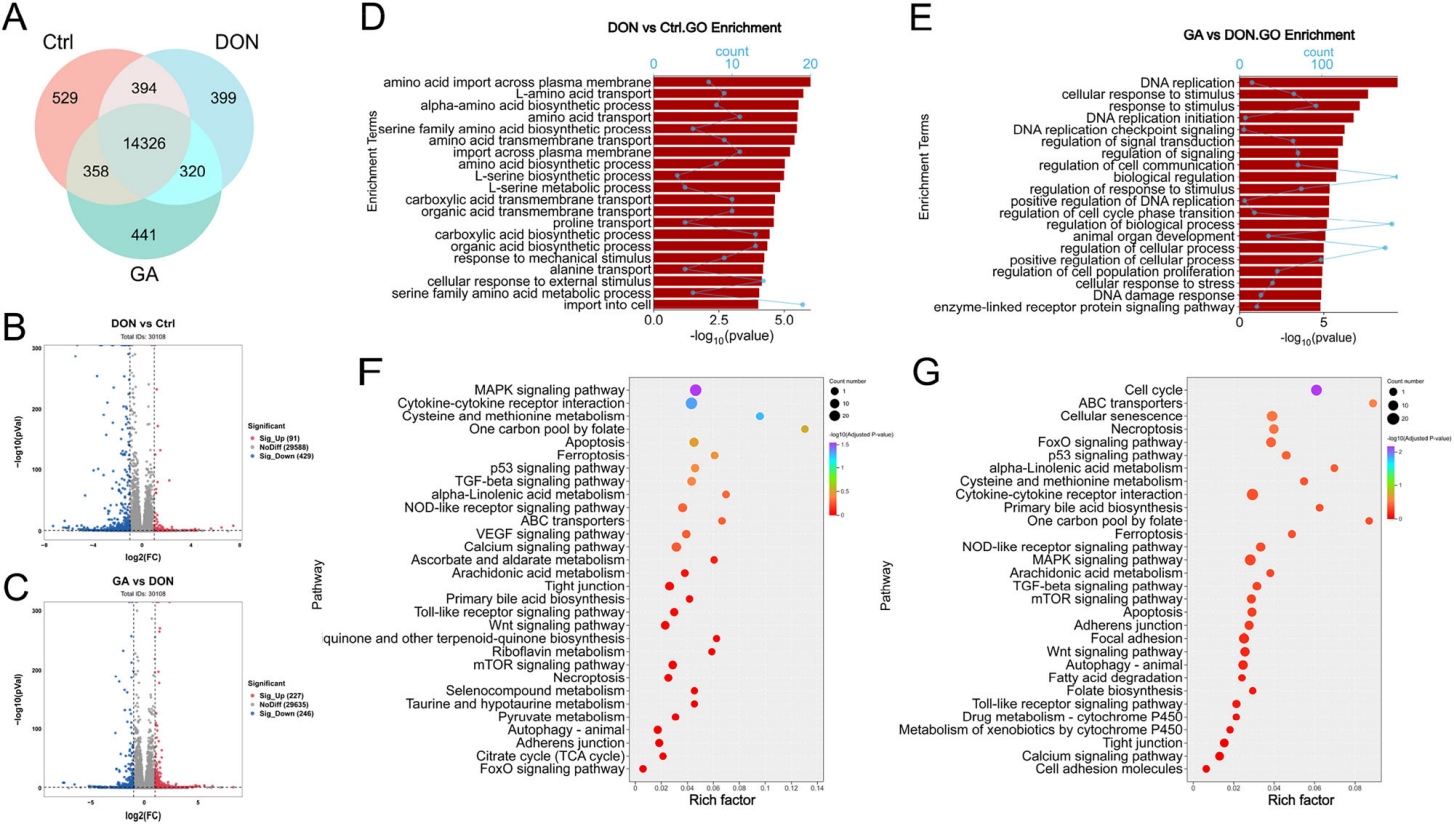
Transcriptomic insights into the mechanisms of GA protection against DON toxicity. **A** Venn diagram of genes detected in the control (Ctrl), DON-treated (DON), and GA-co-treated (GA) groups. Volcano plots visualizing differentially expressed genes (DEGs): **B** DON vs. Ctrl; **C** GA vs. DON. Gene Ontology (GO) enrichment analysis of DEGs: **D** DON vs. Ctrl; **E** GA vs. DON. Kyoto Encyclopedia of Genes and Genomes (KEGG) pathway enrichment analysis of DEGs: **F** DON vs. Ctrl; **G** GA vs. DON.

Transcriptomic profiling was performed to explore the potential mechanism by which GA counteracts DON toxicity in CEF cells. Venn diagram analysis of all sequenced genes showed that the number of genes commonly expressed across the control, DON-treated, and GA-co-treated groups accounted for 94%–97% of the total expressed genes in each group (Fig. 4A). Comparative analysis of these shared genes revealed that DON exposure induced differential expression in 520 genes in CEF cells, with 91 genes up-regulated and 429 genes down-regulated (Fig. 4B). Compared to the DON group, the GA-co-treated group exhibited differential expression of 473 genes, including 227 up-regulated genes and 246 down-regulated genes (Fig. 4C). Following DON treatment of CEF cells, approximately 128 signaling pathways with high Rich Factor values showed significant alterations (Table S2). These pathways were primarily involved in cellular processes such as amino acid metabolism, cellular response to stimuli, transmembrane transport, and other biological processes (Fig. 4D). Based on the Rich Factor, pathway similarity, and impact on cell fate, the top 30 representative pathways were presented in Fig. 4F and Table S3. Compared to the untreated control group, DON treatment altered core pathways related to oxidative stress and ferroptosis in CEF cells, including glutathione metabolism, ferroptosis, and antioxidant metabolism pathways (e.g., ascorbate and aldarate metabolism, riboflavin metabolism); pathways associated with energy metabolism and mitochondrial function (e.g., citrate cycle, pyruvate metabolism, linoleic acid metabolism, mitophagy); amino acid and nucleotide metabolism pathways (e.g., one carbon pool by folate, cysteine and methionine metabolism, purine and pyrimidine metabolism); cell death-related pathways (e.g., apoptosis, necroptosis, and autophagy); immune inflammation and proliferation regulation pathways (e.g., MAPK/p53 signaling, cytokine-cytokine receptor interaction, TLR/NOD pathways, and Wnt signaling); and xenobiotic transport and tissue barrier pathways (e.g., ABC transporters, tight junction, adherens junction, and primary bile acid biosynthesis). Comprehensive analysis of DON-induced differentially expressed genes (DEGs) within these signaling pathways may provide insights into the mechanistic bases of DON-induced damage (Table S3).

Compared to DON treatment alone, co-treatment with GA and DON induced changes in approximately 138 pathways with high Rich Factor values in CEF cells (Table S4). These pathways primarily involved in cellular processes such as DNA replication, cellular response to stimuli, signal transduction, and other biological processes (Fig. 4E). The top 30 representative pathways were selected based on Rich Factor, pathway similarity, and impact on cell fate (Fig. 4G and Table S5). Analysis showed that GA modulated the following pathways: (1) antioxidant defense pathways (e.g., glutathione metabolism, riboflavin metabolism, and ascorbate metabolism); (2) cell death pathways (e.g., ferroptosis, apoptosis, necroptosis, autophagy); (3) energy and metabolism-related pathways (e.g., glycolysis and pentose phosphate pathways, mitochondrial functions like TCA cycle and fatty acid β-oxidation, nucleotide synthesis pathways such as one carbon pool by folate and folate biosynthesis); (4) inflammation and immunomodulation pathways (e.g., NF-κB signaling, NOD-like receptor pathways, cytokine-cytokine receptor interaction); (5) xenobiotic detoxification pathways (e.g., cytochrome P450 systems, including drug metabolism by cytochrome P450 and xenobiotics metabolism, along with ABC transporters); and (6) cell cycle and DNA repair pathways (e.g., DNA damage response, cell cycle regulation). Collectively, these data demonstrate that GA counterbalances the widespread transcriptomic effects of DON, with the most prominent and functionally consistent rescue observed in the ferroptosis pathway. The finding guided our subsequent experiments to validate this specific cell death mechanism.

### GA suppresses ferroptosis to antagonize DON toxicity

**Fig. 5:**
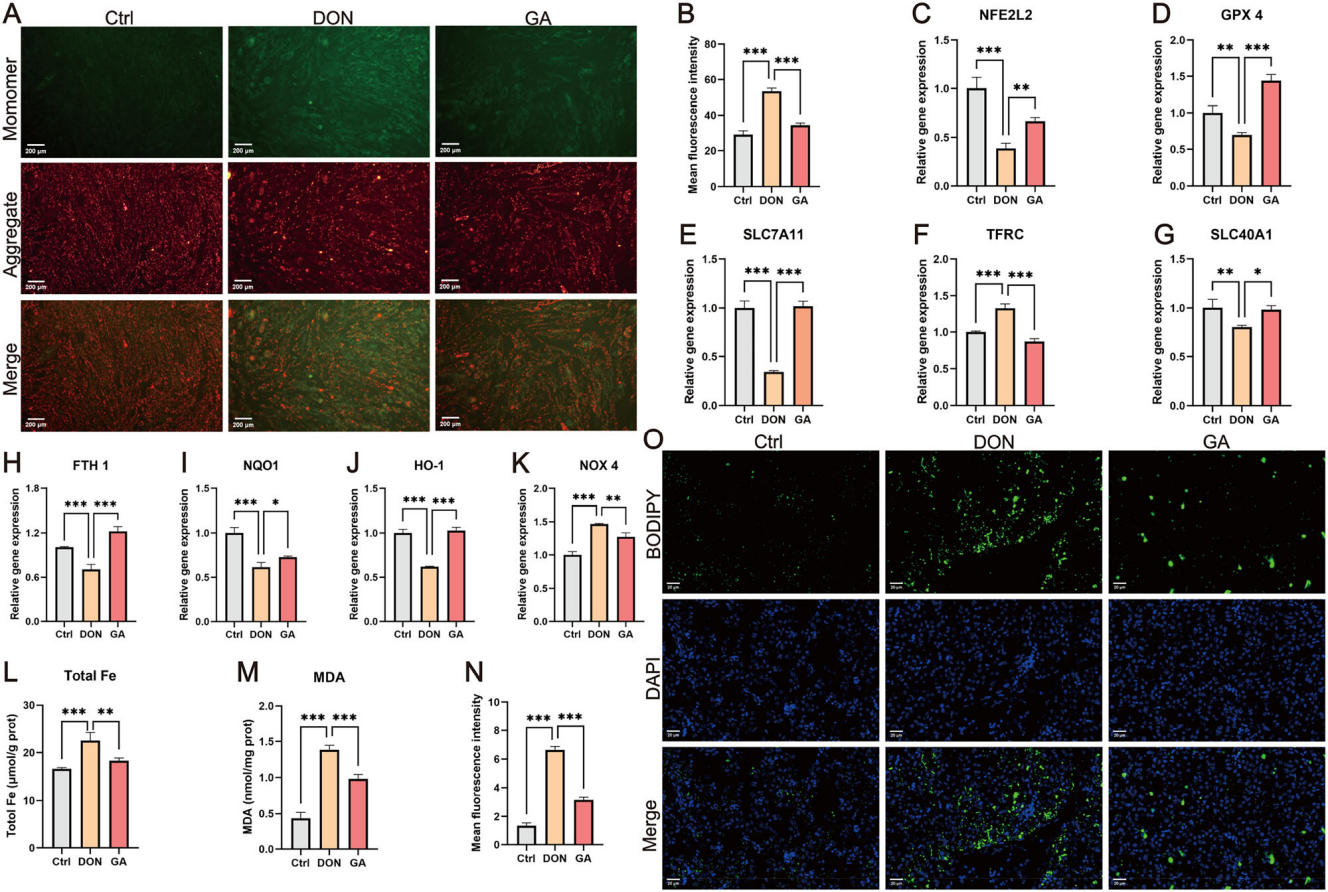
GA alleviates DON toxicity by counteracting DON-induced ferroptosis. **A** JC-1 staining showing dissipation of mitochondrial membrane potential (green: JC-1 monomers; red: JC-1 aggregates; scale bar: 200 μm). **B** Quantitative analysis of green fluorescence intensity in panel A. **C** Expression of *NFE2L2* (the gene encoding Nrf2). Expression of ferroptosis-related genes: *GPX4* (**D**), *SLC7A11* (**E**), *TFRC* (**F**), *SLC40A1* (**G**), *FTH1* (**H**), *NQO1* (**I**), *HMOX1* (encoding HO-1, **J**), and *NOX4* (**K**). **L** Total iron content in liver tissues. **M** Cellular malondialdehyde (MDA) levels. **N** Quantified lipid ROS accumulation (fluorescence intensity of BODIPY™ 581/591 C11). **O** Representative BODIPY™ 581/591 C11 staining (green: oxidized lipids indicating lipid ROS accumulation; blue: DAPI-stained nuclei; scale bar: 20 μm). Data are presented mean ± SD (*n* = 3). Statistical significance: * *p* < 0.05, ** *p* < 0.01, *** *p* < 0.001 (vs. control or DON group).

Transcriptome data suggested that ferroptosis is a potential key pathway linking DON-induced damage and GA-mediated protection. DON exposure led to a significant loss of MMP, indicated by an increase in JC-1 monomer (green) fluorescence (*p* < 0.001). This loss of MMP was mitigated by GA co-treatment (*p* < 0.001) (Fig. 5A, B). Consistent with the transcriptomic data, qPCR analysis confirmed that DON significantly suppressed the expression of *NFE2L2* (the gene encoding nuclear factor erythroid 2-related factor 2, Nrf2) (*p* < 0.001), and this suppression was reversed by GA co-treatment (Fig. 5C). We then detected the expression of ferroptosis-related genes that may be involved in the mechanism of GA action, based on the transcriptome results. Transcriptome-guided analysis confirmed that DON significantly suppressed the expression of anti-ferroptotic genes (*GPX4*, *SLC7A11*, *SLC40A1*, *FTH1*, *NQO1*, *HO-1*; *p* < 0.01) and up-regulated the expression of pro-ferroptotic genes (*TFRC*, *NOX4*; *p* < 0.01). All these DON-induced changes were reversed by GA treatment (Fig. 5D–K). Consistent with the induction of ferroptosis, DON exposure caused tissue iron overload (*p* < 0.001), which was attenuated by GA co-treatment (Fig. 5L). Lipid peroxidation-related markers further confirmed this mechanism: DON elevated malondialdehyde (MDA) levels (a stable end product of lipid peroxidation, *p* < 0.001) and increased lipid ROS accumulation (reflected by green fluorescence intensity of BODIPY™ 581/591 C11, *p* < 0.001), both significantly reduced by GA (Fig. 5M–O). Collectively, these results demonstrate that GA antagonizes DON toxicity by suppressing ferroptosis through multiple pathways.

### Erastin/Fer-1 reversion verifies ferroptosis as fundamental to DON-induced toxicity and GA-mediated antagonism

**Fig. 6:**
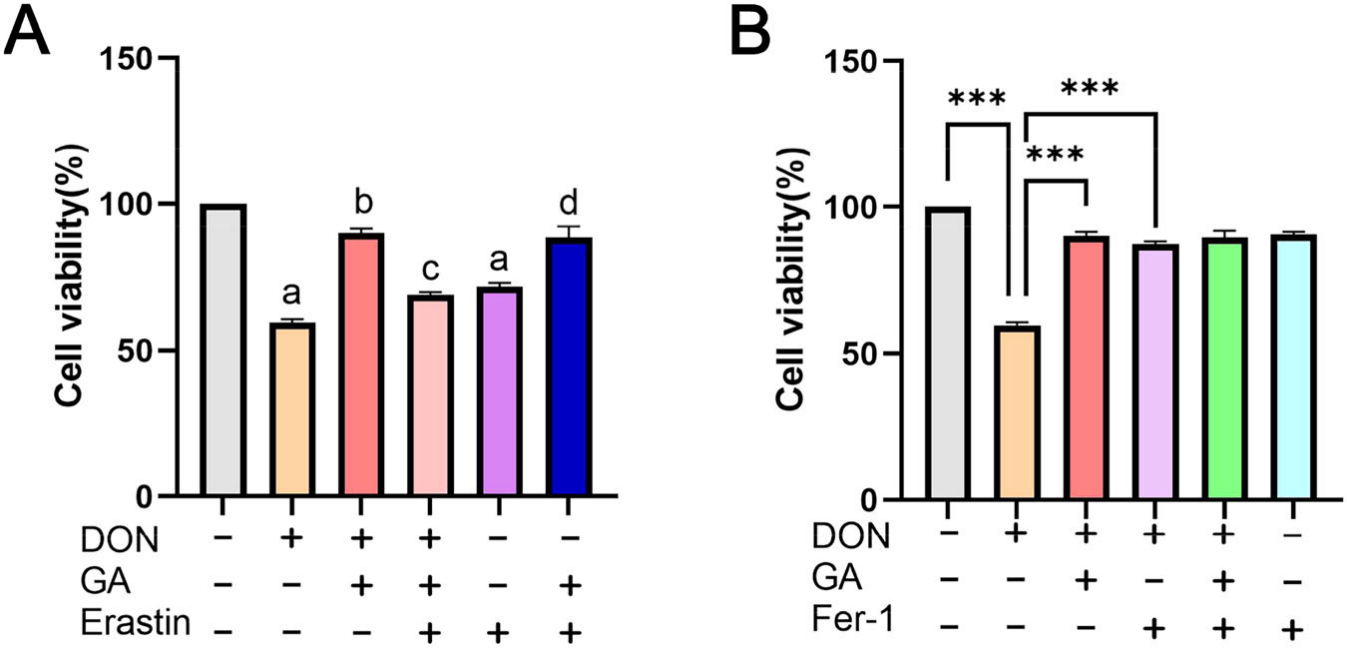
Pharmacological reversion assays confirm the role of ferroptosis in DON-induced toxicity and GA-mediated resistance. **A** Viability of CEF cells treated with DON, GA, and erastin. (a, *p* < 0.05 vs. control (untreated); b, *p* < 0.05 vs. DON; c, *p* < 0.05 vs. DON + GA; d, *p* < 0.05 vs. erastin). **B** Cell viability following treatments with DON, GA, and fer-1. Data are presented as mean ± SD (*n* = 3). Statistical significance: * *p* < 0.05, ** *p* < 0.01, *** *p* < 0.001 (vs. corresponding group).

To pharmacologically validate the involvement of the ferroptosis pathway, we used erastin (a ferroptosis inducer) and ferrostatin-1 (Fer-1, a synthetic ferroptosis inhibitor). A control group without any drug treatment was included. Cell viability assays showed that co-treatment with 160 μM GA significantly rescued CEF cells from erastin-induced cytotoxicity: cell viability increased from 71.81% (erastin alone) to 88.61% (GA + erastin) (*p* < 0.001) (Fig. 6A). This confirms that GA has intrinsic ferroptosis-inhibitory activity. Conversely, supplementing with Fer-1 in DON-treated cells restored cell viability from 59.59% (DON alone) to 87.43% (DON + Fer-1) (*p* < 0.001) (Fig. 6B), directly indicating that ferroptosis is the primary mechanism of DON-induced toxicity. Notably, the DON+ GA + erastin group exhibited significantly lower viability (69.03%) compared to the DON+ GA (90.05%) (*p* < 0.01) (Fig. 6A), demonstrating that erastin overrides the protective effect of GA by reactivating ferroptosis. Importantly, cell viability in the DON+ GA group (90.05%) was comparable to that in the DON + Fer-1 group (87.43%) (Fig. 6A, B), indicating that GA achieves a level of cytoprotection similar to that of Fer-1, canonical ferroptosis inhibitor.

## Discussion

DON is one of the five major agricultural mycotoxins threatening the safe utilization of global staple crops (including corn, barley, and wheat). Humans and animals in grain-growing regions worldwide are chronically exposed to DON, posing significant threats to environmental and food safety^5^. Previous studies have confirmed that DON inhibits chicken growth, causes multi-organ damage, and disrupts intestinal microbiota, thereby impairing production performance^34^. Current approaches for DON detoxification primarily involve physical, chemical, and biological methods^7^. However, complete elimination of DON remains unattainable. Therefore, utilizing natural compounds to counteract tissue and cellular damage induced by DON intake in humans and animals is an indispensable strategy^7^. Among these, natural polyphenols are established to exert multifaceted protective effects on animal cells, including antioxidant and anti-inflammatory activities^7^. Based on the toxicological mechanisms of DON, this study selected several natural polyphenolic compounds with recognized antioxidant properties for preliminary screening. This initial screening pool included gallic acid (GA), caffeic acid (CA), and proanthocyanidins (PC). Among them, CA, a common hydroxycinnamic acid, modulates redox homeostasis and inflammation^38^, while PC are potent free radical scavengers with broad bioactivities^39^. However, under the specific experimental conditions established for DON-induced injury (0.5 μM DON, 9 h exposure), CA and PC exhibited either lower cytoprotective efficacy or significant intrinsic cytotoxicity at effective concentrations (Fig. S1), limiting their suitability for a safe rescue model. Through this comparative assessment, GA was ultimately chosen for in-depth investigation due to its superior combination of potent cytoprotection and negligible intrinsic toxicity. GA, also known as 3,4,5-trihydroxybenzoic acid, is a dietary polyphenol with strong antioxidant activity, widely distributed in various fruits (e.g., pomegranate, caper, grape, hawthorn) and tea^40^. Recent studies have shown that GA exerts a range of biological activities, including anti-inflammatory^41^, anti-cancer^42^, and antioxidant^43^ effects. Furthermore, it has been reported to alleviate specific diseases, such as non-alcoholic fatty liver^44^. This study demonstrated that GA alleviates DON toxicity in chickens at both cellular and animal levels, and explored the specific underlying mechanisms.

This study shows that GA effectively mitigates DON-induced cytotoxicity in chicken embryo fibroblasts. Consistent with existing literature^17,34,45^, DON exposure induced characteristic damage to its primary target organs: the liver and the intestine. As the major site for DON metabolism and detoxification, hepatic injury disrupts critical metabolic functions and systemic homeostasis. Concurrently, damage to the intestinal mucosa—particularly villus shortening and crypt deepening—impairs nutrient absorption and barrier integrity, which are fundamental to poultry growth and health, reflecting a disruption in the normal balance between epithelial cell loss and regeneration. In our chick model, DON indeed induced acute hepatocellular necrosis, inflammation, and elevated serum liver enzyme levels, alongside marked impairment of intestinal villus architecture. Critically, GA intervention significantly mitigated these pathological alterations in both organs, confirming its protective efficacy against DON-induced multi-tissue damage. This hepatoprotective effect of GA is consistent with the documented actions of other phytochemicals that attenuate toxin-induced liver injury via defined intracellular signaling pathways, reinforcing the potential of this compound class in managing mycotoxicosis^46^. DON-induced oxidative stress signaling is considered a key mechanism underlying its induction of DNA damage, protein oxidation, and cell death^14^. Consistent with this, our findings showed that DON induced profound redox imbalance, evidenced by the accumulation of reactive oxygen species (ROS) and the lipid peroxidation product malondialdehyde (MDA), concurrent with depletion of key antioxidants (e.g., glutathione, GSH) and reduced activities of superoxide dismutase (SOD) and catalase (CAT). Crucially, the GA intervention comprehensively restored this balance. This capacity of GA to rescue antioxidant defenses is a consistent effect observed across models of injury induced by various toxicants^47^, highlighting its broad-spectrum antioxidant potency. The effective reversal of these specific oxidative parameters positions the amelioration of oxidative stress not as an isolated effect, but as a fundamental step underpinning GA’s broader protective role. The most significant downstream consequence of rectifying this imbalance appears to be the regulation of ferroptosis, a cell death process intrinsically driven by lipid peroxidation.

Ferroptosis, a novel form of regulated cell death, is characterized by iron-dependent lipid peroxidation, ROS accumulation, and glutathione peroxidase 4 (GPX4) depletion^48^. Recent studies have confirmed that ferroptosis is a critical mechanism of DON toxicity^15,18,49^. Our integrated analyses demonstrate that GA alleviates DON toxicity through a multifaceted inhibition of this cell death pathway. The System Xc⁻/GPX4 pathway, with core genes *GPX4* and *SLC7A11*, is essential for cells to resist lipid peroxide-induced oxidative stress and ferroptosis^50^. Our data showed that DON exposure downregulated *GPX4* and *SLC7A11* expression, which was reversed by GA. Ferritin heavy chain 1 (FTH1) is a component of ferritin, a cytoplasmic iron-storage protein complex capable of chelating up to 4,500 iron atoms, thereby playing a key antioxidant role by sequestering redox-active iron^51^. Transferrin receptor (TFRC) binds to transferrin and is critical for cellular iron uptake^52^, while solute carrier family 40 member 1 (SLC40A1, also known as ferroportin, FPN)—the only known iron exporter in cell membranes—is a key protein inhibiting ferroptosis^53^. DON exposure upregulated *TFRC* and downregulated *FTH1*/*SLC40A1* expression, increasing intracellular iron content and triggering ferroptosis via Fenton reactions, whereas GA reversed these changes to reduce iron overload. NADPH oxidases (NOXs) are major sources of intracellular ROS^54^, while heme oxygenase-1 (HO-1, encoded by *HMOX1*) and NAD(P)H quinone dehydrogenase 1 (NQO1) play pivotal roles in countering oxidative stress and ferroptosis^55,56^. DON exposure upregulated *NOX* family members, downregulated *HO-1*/*NQO1* expression, and increased ROS production to induce ferroptosis, whereas GA treatment suppressed *NOX* expression, upregulated *HO-1*/*NQO1* expression, and alleviated ferroptotic cell death. Notably, ferroptosis induction appears to be a convergent mechanism for DON and other chemically distinct hepatotoxins^57^. In conclusion, GA mitigates DON toxicity by reversing DON-induced antioxidant imbalance, iron overload, and excessive ROS production, thereby inhibiting ferroptosis.

The transcription factor nuclear factor erythroid 2-related factor 2 (Nrf2, encoded by *NFE2L2*) serves as a key regulator of redox balance, metabolism, protein homeostasis, and inflammation. Its expression level is directly associated with ferroptosis sensitivity: increased Nrf2 expression inhibits ferroptosis, while decreased expression enhances the sensitivity of cancer cells to ferroptosis inducers^58,59^. Studies have shown that Nrf2 regulates numerous key genes involved in iron metabolism, antioxidant systems, and lipid metabolism, thereby modulating ferroptosis^60^. Consistent with this central regulatory role, it was found that GA counteracted DON-induced suppression of Nrf2 expression, which aligns with its known Nrf2-activating properties^27,61,62^. The functional significance of this activation is underscored by its downstream targets. As Nrf2 target genes, *HO-1* and *NQO1* have been proven to protect cells and inhibit ferroptosis upon Nrf2 activation^63,64^. In ethanol-induced gastric ulcers, a strong negative correlation was found between Nrf2 and NOXs, particularly NOX4^65^. Neferine regulates iron metabolism via the Nrf2/FPN axis and exerts antioxidant effects through the Nrf2/System Xc⁻/GPX4 axis in mice with severe acute pancreatitis (SAP), thereby inhibiting ferroptosis and exerting protective effects^66^. In Nrf2 knockout (KO) cells, *TFRC* mRNA expression is decreased, increasing cellular sensitivity to ferroptosis^67^. *FTH1*, a target gene of Nrf2, is transcriptionally regulated by Nrf2^68^. Thus, this study infers that GA alleviates DON toxicity by activating Nrf2 expression, thereby inhibiting DON-induced ferroptosis in chicken embryo fibroblasts and chicks. Our use of ferroptosis inducer and inhibitor Fer-1 further validates the involvement of ferroptosis in this process.

Beyond the core ferroptosis pathway, our transcriptomic analysis suggests that DON toxicity and GA protection involve auxiliary adjustments across interconnected metabolic networks, which collectively shape cellular susceptibility to ferroptosis. Specifically, DON disrupted glutathione metabolism by downregulating genes critical for precursor synthesis [e.g., cystathionine gamma-lyase (CTH)^69^ and cystathionine beta-synthase (CBS)^70^] and compartmentalized redox balance [e.g., ATP-binding cassette subfamily B member 10 (ABCB10)^71^]. GA counteracted this by modulating genes involved in glutathione regeneration [e.g., methionine adenosyltransferase 2A (MAT2A)^72^] and precursor conservation. Concurrently, DON impaired transmembrane transport and lipid homeostasis by suppressing genes such as ATP-binding cassette subfamily A member 4 (ABCA4)^73^, whereas GA induced transporters for detoxification and lipid efflux (e.g., ABCB11^74^ and ABCA1^75^). Perturbations in metal ion homeostasis were also prominent. DON suppressed genes facilitating iron metabolism[e.g., six-transmembrane epithelial antigen of prostate 1/2 (STEAP1/2)^76^ and hepcidin antimicrobial peptide (HAMP)^77^], potentially promoting iron retention. In contrast, GA elevated expression of the zinc transporter solute carrier family 39 member 10 (SLC39A10)^78^, which may competitively inhibit iron uptake. Furthermore, DON-induced downregulation of apoptotic effectors caspase 3 (CASP3) and caspase 10 (CASP10)^79^ aligns with a shift toward ferroptosis. Collectively, these transcriptional alterations indicate that GA orchestrates a systemic cytoprotective program, rectifying iron dyshomeostasis, antioxidant deficits, and metabolic disruption to enhance cellular resilience against ferroptotic damage.

## Conclusion

In summary, this study demonstrates that GA protects against DON-induced toxicity primarily by activating the Nrf2 pathway and inhibiting ferroptosis. Our findings validate a mechanism-targeted screening strategy, successfully identifying GA as an effective antagonist against the defined toxic pathway (ferroptosis/oxidative stress). However, the protective effect of a single compound is inherently limited. This limitation may stem from the compound’s own efficacy, but more importantly, it reflects the multifaceted and incompletely understood nature of DON’s toxicological mechanisms. Therefore, a more promising future direction resides in developing and applying a higher-dimensional, multi-omics-guided, multi-target intervention strategy. Systematically profiling the perturbations induced by DON exposure, including ubiquitination, phosphorylation, and other post-translational modifications, alongside genomic, epigenomic, and metabolomic alterations, will be crucial to mapping its comprehensive toxicological network and identifying additional key pathogenic nodes. Correspondingly, this will enable the screening or design of compounds targeting these distinct nodes. Ultimately, the rational combination of mechanism-specific protectants—such as GA targeting ferroptosis with compounds counteracting other key injuries (e.g., inflammation or barrier dysfunction)—holds the greatest potential for creating a synergistic and robust defense against the complex pathology of DON.

### Supplementary materials

The following supporting information can be downloaded at: www.xxx.com/xxx/s1, Supplementary Table 1: Primers used for the quantitative real-time PCR; Supplementary Table 2: KEGG Signaling Pathways Altered by DON Exposure; Supplementary Table 3: Top 30 Representative Signaling Pathways Altered by DON Exposure and Their Differentially Expressed Genes; Supplementary Table 4: KEGG Signaling Pathways Altered by GA and DON Co-Treatment; Supplementary Table 5: Top 30 Representative Signaling Pathways Altered by GA Co-Treatment and Their Differentially Expressed Genes; Supplementary Fig. 1: CA and PC Mitigate DON-induced Cytotoxicity in CEF cells; Supplementary Fig. 2: H&E Staining of Duodenum and Jejunum in Ctrl, DON, and GA Groups.

## Supplementary information

Supplemental Materials LWT

Table S2. KEGG Signaling Pathways Altered by DON Exposure

Table S3. Top 30 Representative Signaling Pathways

Table S4. KEGG Signaling Pathways

Table S5. Top 30 Signaling Pathways Altered by GA

## Data Availability

Data will be made available on request.
