# Supplementary material for: Gallic acid antagonizes deoxynivalenol toxicity by inhibiting DON-induced ferroptosis
Source: NPJ Sci Food. 2026 Mar 5;10:127. doi: 10.1038/s41538-026-00782-y (PMC13076626; doi:10.1038/s41538-026-00782-y)
Supplement: Supplementary file 1 — Supplemental Materials LWT [file 41538_2026_782_MOESM1_ESM.docx]

Supplemental Materials for

**Gallic acid antagonizes deoxynivalenol toxicity by inhibiting DON-induced ferroptosis**

Haorong Wang ^1,2,3,5^, Jiacui Xu^1,2,3,5^, Jinghan Feng^1,2,3^, Yifan Zhang^1,2,3^, Zixian Zhao^1,2,3^, Yan Lv^1,3^, Chuanqi Wang^1,2^, Jing Zhang^1,2,4^🖂,Yongxing Ai^1,2,4^🖂

^1^ College of Animal Science, Jilin University, 5333 Xi’An Road, Changchun 130062, Jilin, China

^2^ Jilin Provincial Key Laboratory of Livestock and Poultry Feed and Feeding in Northeastern Frigid Area, 5333 Xi’An Road, Changchun 130062, Jilin, China

^3^ State Key Laboratory for Diagnosis and Treatment of Severe Zoonotic Infectious Diseases, College of Veterinary Medicine, Jilin University, 5333 Xi’An Road, Changchun 130062, Jilin, China

^4^Institute of Food Quality and Nutrition, Academy of National Food and Strategic Reserves Administration, Beijing 100037, China

^5^These authors contributed equally: Haorong Wang; Jiacui Xu (ORCID ID: 0000-0002-6454-4440).

🖂e-mail: aiyx@jlu.edu.cn (Y.A.) (ORCID ID:0000-0001-5212-3609); zj@ags.ac.cn(J.Z.)

List of contents:

Supplementary Table 1

Supplementary Figure 1 to 2

Table S1. Primers used for the quantitative real-time PCR

| Gene name | Accession Number | Sequence of primers (5’-3’) |
| --- | --- | --- |
| β-actin | [**NM_205518.2**](https://www.ncbi.nlm.nih.gov/nuccore/NM_205518.2) | F: GTGATGGACTCTGGTGATG |
|  |  | R: GCGTAGCCTTCATAGATGG |
| Nrf2 | [**NM_001396902.1**](https://www.ncbi.nlm.nih.gov/nuccore/NM_001396902.1) | F: TAACGATGACCACTCAGGAACT |
|  |  | R: CCACAGGTAGATGATTCAACAGAG |
| GPX4 | [**NM_001346448.2**](https://www.ncbi.nlm.nih.gov/nuccore/NM_001346448.2) | F: CACCTCCATCTACGACTTCCA |
|  |  | R: AGATCGACGAGCTGAGTGTAAT |
| SLC7A11 | [**XM_426289.7**](https://www.ncbi.nlm.nih.gov/nuccore/XM_426289.7) | F: ACTGGTAGTTGCTGGCTTGA |
|  |  | R: ATGAAGAGGCAGGTGAAGGAA |
| TFRC | [**NM_205256.2**](https://www.ncbi.nlm.nih.gov/nuccore/NM_205256.2) | F: AAGTCAGAGGCAGCACCAA |
|  |  | R: GCAACGATAGCATCAGGAGTC |
| SLC40A1 | [**NM_001012913.2**](https://www.ncbi.nlm.nih.gov/nuccore/NM_001012913.2) | F: GGTTCTGTGCTAAGCCTCCT |
|  |  | R: TGCGACGAAGCCAAGTGA |
| FTH1 | [**NM_205086.2**](https://www.ncbi.nlm.nih.gov/nuccore/NM_205086.2) | F: TGACCAACCTGCGGAAGATG |
|  |  | R: GGTACTCTGCCATGCCATACT |
| NQO1 | [**NM_001277619.2**](https://www.ncbi.nlm.nih.gov/nuccore/NM_001277619.2) | F: CCCGAGTGCTTTGTCTACGA |
|  |  | R: GGTCAGCCGCTTCAATCTTC |
| HO-1 | [**NM_205344.2**](https://www.ncbi.nlm.nih.gov/nuccore/NM_205344.2) | F: AAGGAAGCCACCAAGGAAGT |
|  |  | R: CCTCCAGAGCAGAGTAGATGAA |
| NOX4 | [**NM_001101829.3**](https://www.ncbi.nlm.nih.gov/nuccore/NM_001101829.3) | F: TACCAGACCAACTTAGAGGAACA |
|  |  | R: AAGGCTCAGTAGTATAGTCAGGAA |

F=Forward primer; R=Reverse primer

Figure S1


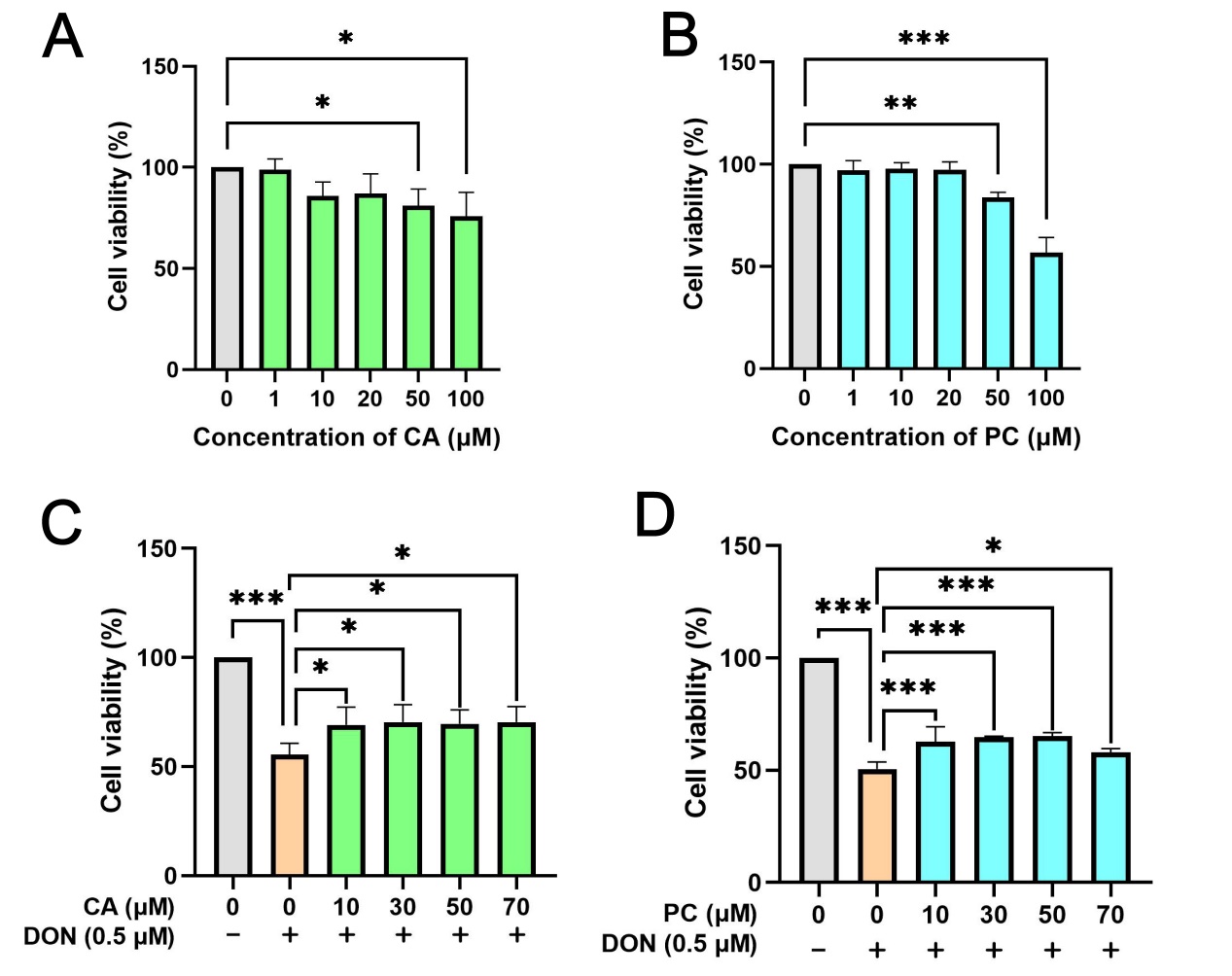


Figure S1. CA and PC Mitigate DON-induced Cytotoxicity in CEF cells.

(A) Cell viability after CA treatment (1-100 μM). (B) Cell viability after PC treatment (1-100 μM). (C) Cell viability after co-treatment with CA (10–70 μM) and 0.5 μM DON. (D) Cell viability after co-treatment with PC (10-70 μM) and 0.5 μM DON. Error bars represent the standard deviations from three repeated measurements. **p*<0.05, ** *p*<0.01, *** *p*<0.001.

Figure S2


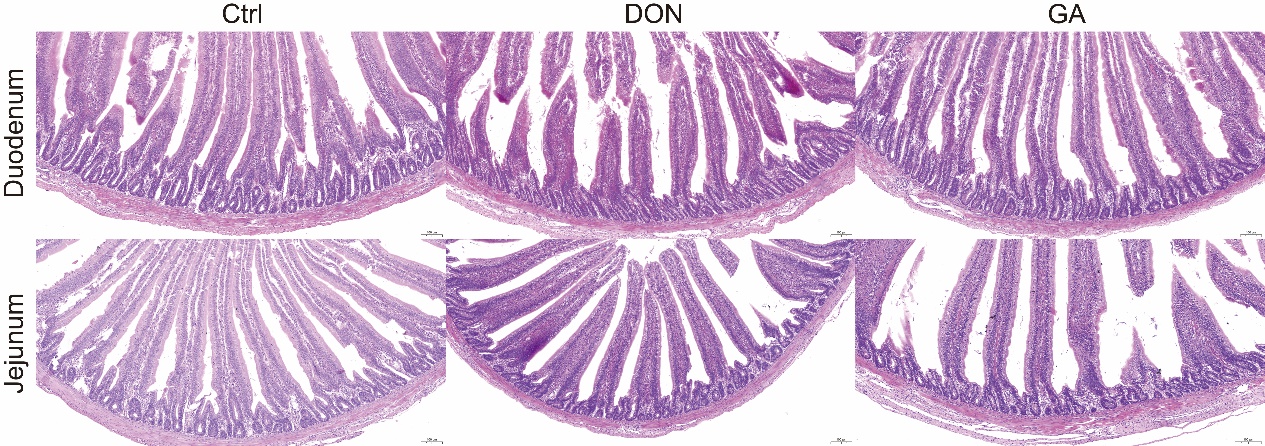


Figure S2. H&E Staining of Duodenum and Jejunum in Ctrl, DON, and GA Groups.
